# Sacral Fractures Are Increasing in Frequency, with Females and Falls Comprising the Majority: A NEISS Cross-Sectional Study from 2003 to 2022

**DOI:** 10.3390/jcm15207895

**Published:** 2026-10-13

**Authors:** Favour C. Ononogbu-Uche, William J. Bohne, Arnav Sharma, Arianna Brinkley, Andrii A. Del Rosario, Karim Akl, Richard Jiang, Harry Wang, Andrew W. Froehle, Allen A. Champagne, Ethan Cottrill, William Richardson, Malcolm DeBaun, Norah A. Foster, Muhammad M. Abd-El-Barr

**Affiliations:** 1 College of Medicine, SUNY Downstate Health Sciences University, Brooklyn, NY 11203, USA; favour.ononogbu-uche@downstate.edu (F.C.O.-U.); andriimigu.delrosario@downstate.edu (A.A.D.R.); 2 Department of Orthopedic Surgery, Boonshoft School of Medicine, Wright State University, Dayton, OH 45409, USA; bohne.3@wright.edu (W.J.B.); andrew.froehle@wright.edu (A.W.F.); nafoster@premierhealth.com (N.A.F.); 3 Department of Neurosurgery, Duke University, Durham, NC 27710, USA; arnav.sharma@duke.edu (A.S.); arianna.brinkley@duke.edu (A.B.); kareemakl2004@gmail.com (K.A.); rjiang711@gmail.com (R.J.); wangh1222@gmail.com (H.W.); 4 Department of Orthopedic Surgery, Duke University, Durham, NC 27710, USA; allen.champagne@duke.edu (A.A.C.); ethan.cottrill@duke.edu (E.C.); william.richardson@duke.edu (W.R.); malcolm.debaun@duke.edu (M.D.)

**Keywords:** sacral fracture, trauma, epidemiology, falls, emergency department, disposition

## Abstract

**Background/Objectives:** Sacral fractures are clinically important injuries that may be occult at presentation and may occur after both low-energy falls and higher-energy trauma. National emergency department data describing temporal trends, mechanisms, associated fractures, and disposition remain limited. **Methods:** A retrospective cross-sectional study was performed using the National Electronic Injury Surveillance System from 2003 to 2022. Sacral fracture emergency department visits were identified using diagnosis and body-part codes with narrative confirmation of sacral involvement. National estimates were generated using NEISS survey weights. The primary outcome was population-based ED visits per 100,000 United States population, overall and stratified by sex. Secondary outcomes included mechanism category, injury location, concurrent vertebral or hip fracture, and emergency department disposition. **Results:** A total of 1747 sacral fracture emergency department visits were identified, representing an estimated 72,215 national visits. Low-energy falls accounted for 67.9% of presentations. Mean age was 61.1 years, and 70.0% of patients were female. Injuries most commonly occurred at home (50.5%). Annual ED visit rate increased from 2003 to 2022, with a steeper rise among females than males. Disposition varied by mechanism. Motor vehicle or transport-related injuries had the highest admission or hospitalization proportion (54.6%), whereas high-energy falls had the highest treated-and-released proportion (62.0%). In survey-aware multinomial regression, increasing age was associated with lower odds of high-energy falls, motor vehicle or transport injuries, and non-fall other injuries compared with low-energy falls. Male sex was associated with motor vehicle or transport mechanism. **Conclusions:** National emergency department presentations for sacral fractures increased from 2003 to 2022 and were dominated by low-energy falls among older female patients. However, transport-related and higher-energy mechanisms showed distinct demographic and disposition patterns. These findings supported trauma-system awareness, mechanism-informed co-injury screening, and orthopedic or spine referral pathways for sacral fracture presentations.

## 1. Introduction

Sacral fractures (SFx) caused substantial pain, impaired mobility, and functional decline, but were frequently occult or delayed in diagnosis when symptoms were nonspecific and first-line imaging was insensitive [1]. The emergency department (ED) was often the first setting in which SFx required suspicion, evaluation, and triage [2]. SFx spanned low-energy fragility-associated injuries and higher-energy traumatic injuries [3]. In older adults, standing-height falls could precipitate sacral insufficiency fractures in the setting of diminished bone quality, whereas younger patients more often sustained SFx after high-energy falls, crush mechanisms, or transport-related trauma [4]. SFx could coexist with vertebral, pelvic ring, or hip fractures, complicating diagnosis, management priorities, and recovery [5]. These features made SFx relevant to ED triage, imaging decisions, mobility assessment, and disposition planning [6]. Population-level ED data on SFx remained limited because prior literature relied largely on single-center series, inpatient datasets, or specialty registries, which may underrepresent less severe or nonoperatively managed injuries [7]. National ED surveillance offered an opportunity to characterize acute-care burden, injury context, and triage patterns in real-world presentations [8]. Therefore, this study sought to characterize national emergency department presentations for sacral fractures from 2003 to 2022 and tested the hypothesis that ED visit rate, demographics, disposition, and injury context differed across mechanism categories.

## 2. Materials and Methods

### 2.1. Data Source, Case Identification, and Variable Classification

**Figure 1:**
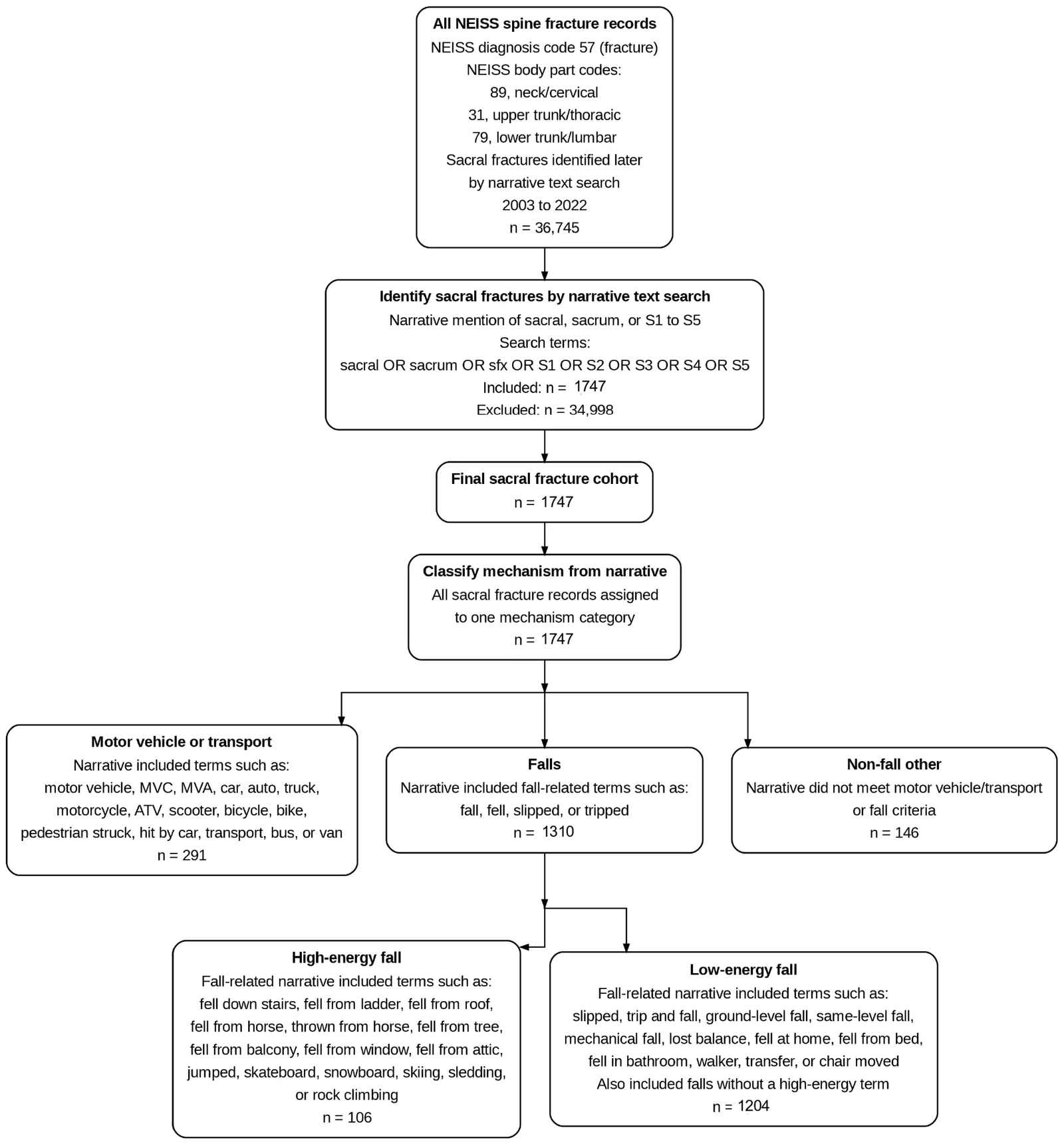
Sacral fracture cohort derivation and mechanism classification.

A retrospective cross-sectional study was performed using the National Electronic Injury Surveillance System (NEISS), a United States Consumer Product Safety Commission database that samples more than 100 emergency departments in the United States and its territories to generate nationally representative estimates of consumer product-related injury visits. NEISS data are publicly available and de-identified, so institutional review board approval was not required. Candidate records were first identified from all NEISS spine fracture records from 2003 to 2022 using diagnosis code 57 (fracture) and body-part codes 89 (neck/cervical), 31 (upper trunk/thoracic), and 79 (lower trunk/lumbar). Sacral fractures were then identified by manual narrative review. Narratives were required to include sacral-specific terms, such as “sacral,” “sacrum,” or sacral level terms including “S1” through “S5,” together with fracture terminology. Because NEISS does not contain a dedicated sacral fracture body-part code, this strategy was designed to identify candidate spine-fracture records for subsequent narrative confirmation. Records describing non-sacral pelvic fractures, isolated coccygeal injuries, or vertebral fractures without sacral involvement were excluded. Narrative review was performed by independent dual review using prespecified inclusion and exclusion criteria. After narrative review, non-pertinent records were excluded, yielding a final analytic cohort of unweighted sacral fracture emergency department visits. This workflow is depicted in Figure 1. Variables included age, analyzed continuously and in 10-year intervals, and sex, reported as male or female. Mechanism was classified into four clinically interpretable categories using NEISS product codes, setting variables, and narrative keywords: low-energy fall, high-energy fall, motor vehicle/transport, and non-fall other. Injury location used NEISS location codes; concurrent vertebral or hip fractures required narrative confirmation, and ED management used the disposition variable. Concurrent fractures were classified into four mutually exclusive categories based on narrative review: vertebral fracture only, hip fracture only, both vertebral and hip fractures, or neither. Cases with both injuries were assigned exclusively to the “both vertebral and hip fractures” category and were not additionally counted within the vertebral-only or hip-only categories.

### 2.2. Outcome Measures and Statistical Analysis

The annual population-based emergency department visit rate was calculated by dividing the yearly weighted national estimate of sacral fracture emergency department visits by the corresponding U.S. population denominator and multiplying by 100,000. Sex-stratified and age-stratified rates used the corresponding sex-specific and age-specific population denominators. Mean age for the overall cohort was estimated as a weighted mean. For descriptive comparisons across mechanism groups, categorical variables were evaluated using survey design-adjusted chi-square testing with Bonferroni correction for multiple comparisons, and continuous variables were evaluated using survey-weighted regression or rank-based comparisons, as appropriate. Unweighted case counts were reported to describe the analytic sample, whereas weighted estimates, percentages, ED visit rates, and regression coefficients were used for national inference. Descriptive estimates were weighted to generate nationally representative estimates. Survey-aware regression analyses incorporated the NEISS complex survey design, with unequal-probability NEISS sampling weights used so coefficient estimates represented national emergency department visits rather than the raw sample, and with NEISS stratum and primary sampling unit identifiers incorporated for design-based variance estimation.

Temporal trends in ED visit rates were evaluated by first deriving yearly sex-specific weighted national estimates and design-based standard errors, then modeling using inverse-variance weighted linear regression with sex, mean-centered calendar year, and a sex-by-year interaction. Age trends were evaluated using survey-weighted linear regression. Mechanism category was modeled using survey-aware multinomial logistic regression with age per 10 years, sex, and mean-centered calendar year as predictors, using low-energy falls as the reference. Multinomial inference used design-based sandwich variance estimation, exponentiated Wald confidence intervals, and Wald z-tests. The ED visit rate model R^2^ reflected fit to annual aggregate estimates. Analyses were performed in R.

## 3. Results

### 3.1. Baseline Characteristics and Injury Mechanisms

The study identified 1747 unweighted sacral fracture (SFx) emergency department visits, corresponding to an estimated 72,215 weighted national visits. Overall, the mean patient age was 61.1 years (SD 23.7), 70.0% were female, and 73.1% were White, with race unspecified in 17.8% of visits. Low-energy falls accounted for most SFx presentations (67.9%), followed by motor vehicle or transport-related injuries (17.2%), non-fall other mechanisms (8.5%), and high-energy falls (6.4%). By etiology, low-energy falls remained the largest subgroup, comprising 1210 unweighted cases and 49,011 weighted visits, followed by motor vehicle or transport injuries (291 unweighted, 12,446 weighted), non-fall other mechanisms (146 unweighted, 6106 weighted), and high-energy falls (106 unweighted, 4653 weighted) (Table 1).

**Figure 2:**
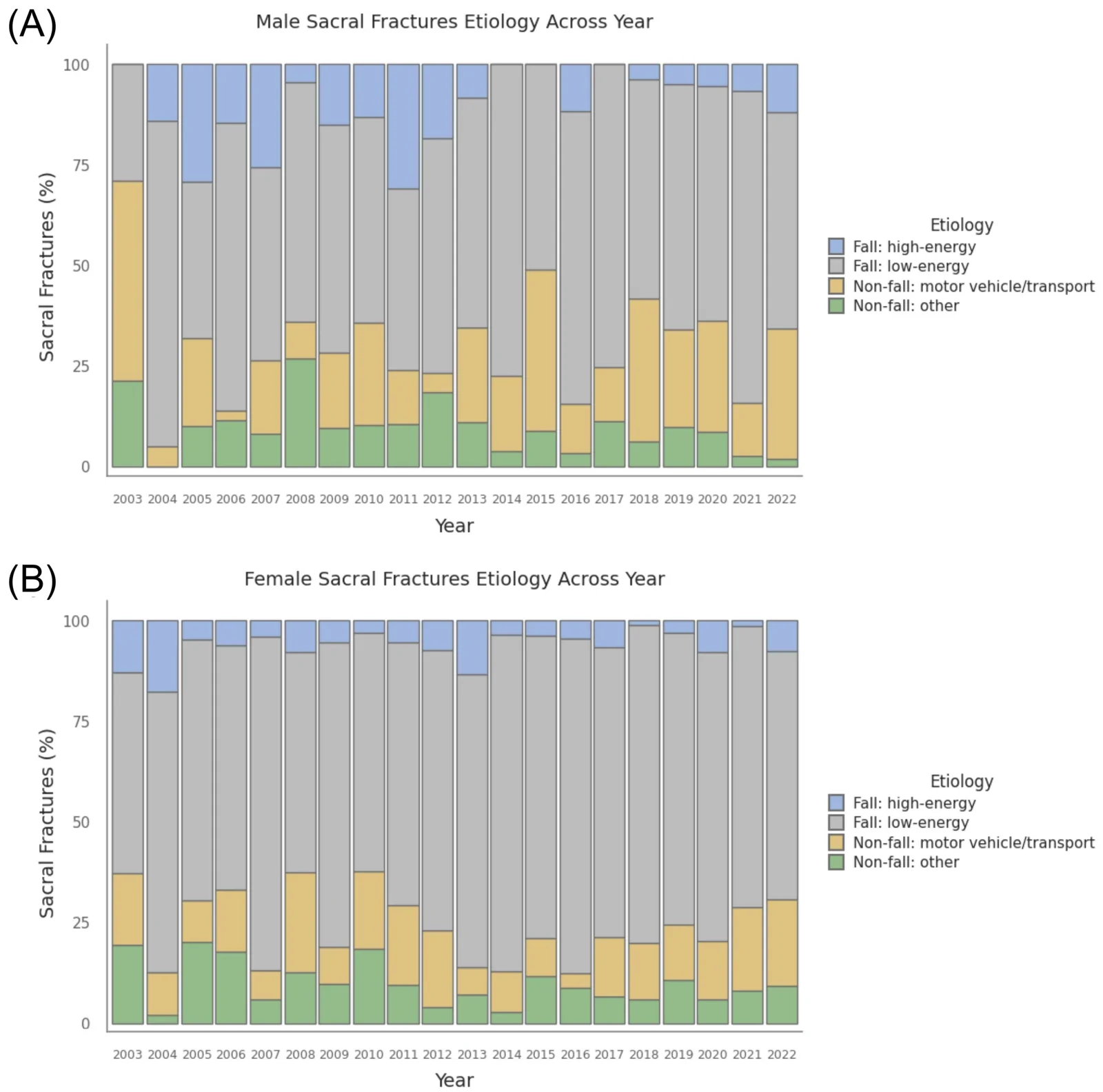
Stacked bar graphs depicting the yearly percent distribution of sacral fractures by etiology from 2003 to 2022 in (**A**) males and (**B**) females.

Demographic characteristics varied significantly across etiologies. Age differed by mechanism (*p* < 0.001), with patients sustaining high-energy falls being the youngest on average (44.9 years, SD 20.7), followed by those with motor vehicle or transport injuries (52.4 years, SD 24.0) and non-fall other mechanisms (54.7 years, SD 25.0), while patients with low-energy falls were the oldest (65.7 years, SD 22.2). Sex distributions also differed significantly (*p* < 0.001). High-energy falls and motor vehicle or transport injuries were relatively more male-represented (41.1% and 40.3% male, respectively), whereas low-energy falls and non-fall other mechanisms were predominantly female (73.2% and 73.6%, respectively). In contrast, race distributions did not differ significantly across etiologies (*p* = 0.126). White patients comprised the majority in every subgroup, ranging from 71.1% in motor vehicle or transport injuries to 75.1% in non-fall other mechanisms, while race was unspecified in 16.5% to 18.1% of cases across groups. Overall, the cohort was characterized by an older, female-predominant, low-energy fall phenotype, with significant variation in age and sex, but not race, across mechanism groups. Figure 2A,B illustrate etiology distributions over time and by sex (Table 2).

### 3.2. Increasing Sacral Fracture ED Visits and Age at Injury

**Figure 3:**
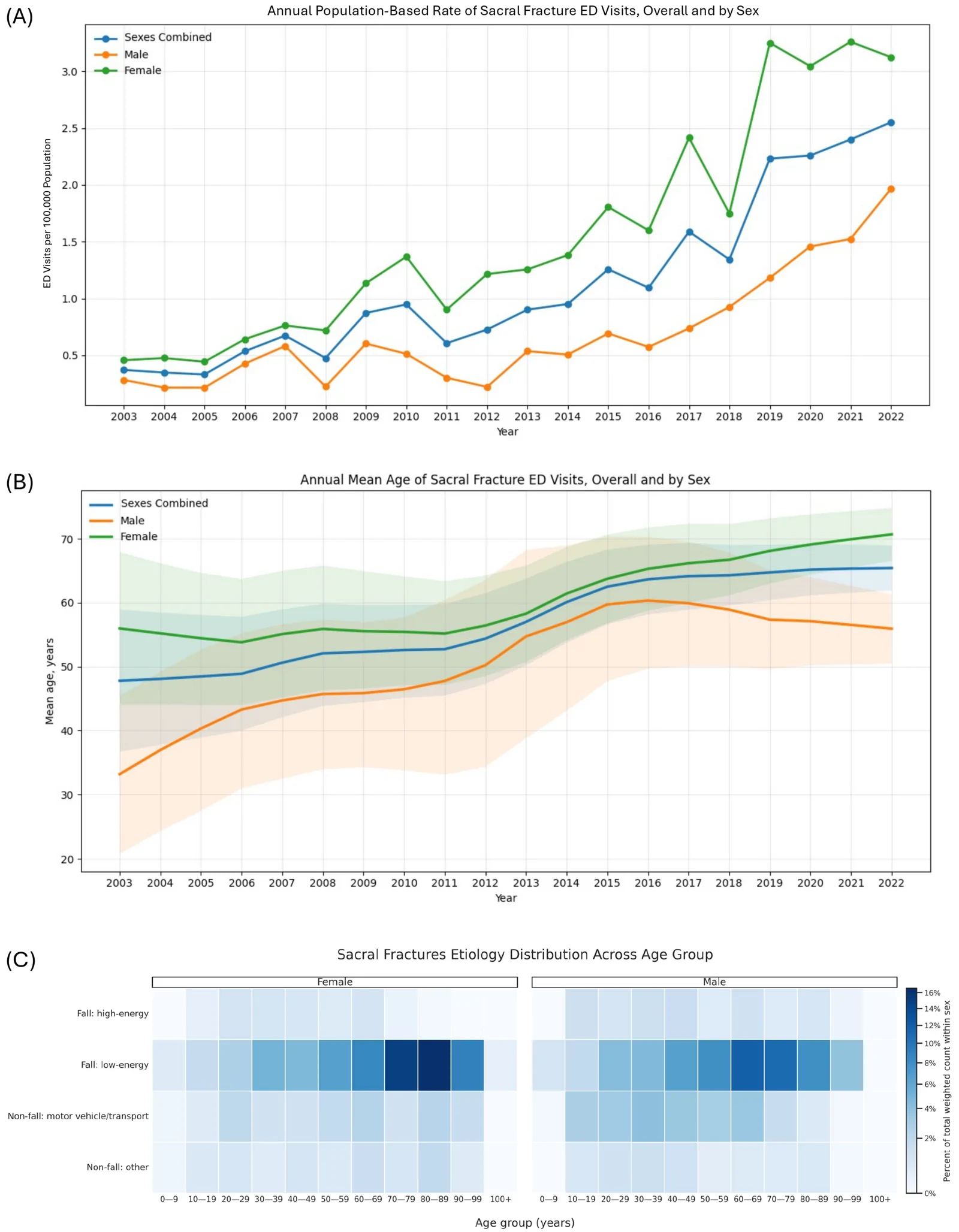
(**A**) Annual population-based emergency department (ED) visit rates of sacral fractures per 100,000 population, overall and stratified by sex, from 2003–2022. (**B**) Annual mean age at ED presentation for sacral fractures, overall and stratified by sex, with shaded regions representing 95% confidence intervals. (**C**) Heatmap of weighted male and female sacral fracture subpopulations by etiology and age group, with darker shades indicating higher proportions of the total weighted count within each sex.

In survey-aware analyses of annual sacral fracture trends, both ED visit rate and patient age increased over time, with important differences by sex. The annual ED visit-rate model used yearly sex-specific national estimates derived with NEISS weights, strata, and primary sampling units and then modeled these estimates using inverse-variance weighted regression. In this model, females had a higher ED visit rate than males at the mean study year by 0.857 per 100,000 population (95% CI, 0.664–1.050; *p* < 0.001). Among males, ED visit rate increased by 0.039 per 100,000 per year (95% CI 0.023 to 0.055, *p* < 0.001). This increase was steeper among females, with an additional female-by-year effect of 0.072 per 100,000 per year (95% CI 0.041 to 0.103, *p* < 0.001), corresponding to an estimated female slope of 0.111 per 100,000 per year (95% CI 0.084 to 0.138, *p* < 0.001; R^2^ = 0.800) (Figure 3A). In a survey-weighted linear model of age using NEISS weights, strata, and PSU, both calendar year and sex were independently associated with age. Mean age increased by 1.05 years per year (95% CI 0.75 to 1.36, *p* < 0.001), and females were older than males by 10.46 years on average (95% CI 7.58 to 13.33, *p* < 0.001; R^2^ = 0.688) (Figure 3B). Consistent with these findings, women presented at older ages overall than men, with mean ages of 64.2 years (SD 23.4) and 53.9 years (SD 22.8), respectively. Among females, sacral fractures were concentrated at older ages and were predominantly driven by low-energy falls, with low-energy fall cases at ages 70 to 99 accounting for 40.6% of the total female sacral fracture burden (15.0% at ages 70 to 79, 16.5% at ages 80 to 89, and 9.1% at ages 90 to 99). In contrast, males showed a broader age distribution, with low-energy falls at ages 60 to 69 and 70 to 79 accounting for 11.8% and 11.0% of the total male burden, respectively, and motor vehicle or transport injuries at ages 10 to 39 accounting for a combined 10.3% of the total male burden (Figure 3C).

### 3.3. Setting, Concurrent Fractures, and Emergency Department Disposition

Across the full SFx cohort of 1747 unweighted cases, corresponding to 72,215 weighted national visits, injuries most often occurred at home (50.5%), followed by other public property (10.9%), recreation or sports settings (7.7%), and streets or highways (5.7%), although location was unspecified in 24.3% of cases. Concurrent fractures were also common: vertebral fractures were present in 21.9% of cases, hip fractures in 10.6%, and both vertebral and hip fractures in 2.1%, while 69.5% involved neither. Emergency department disposition was divided primarily between treated or examined and released (46.7%) and treated and admitted or hospitalized (45.7%), with smaller proportions treated and transferred (4.9%), held for observation (2.5%), or leaving without being seen (0.1%) (Table 3).

**Figure 4:**
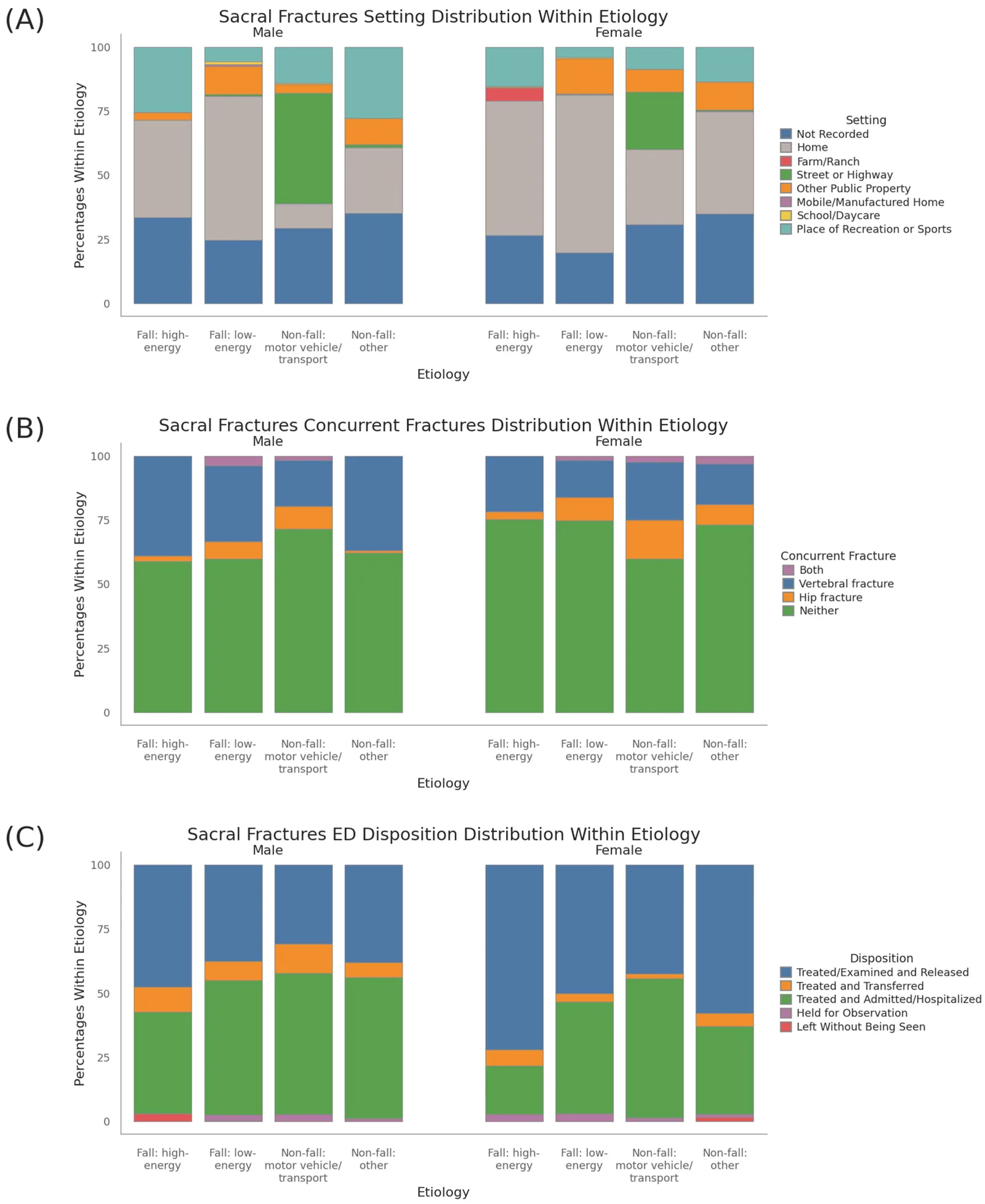
Stacked bar graphs depicting the breakdown of various metrics across injury etiology and separated by sex: (**A**) setting, (**B**) concurrent fractures, and (**C**) emergency department disposition.

When stratified by etiology, both injury setting and disposition differed significantly across mechanism groups (setting *p* < 0.001, disposition *p* = 0.01), whereas concurrent fracture patterns did not (*p* = 0.167) (Table 4). Low-energy falls occurred most often at home (60.1%), motor vehicle or transport injuries occurred most often on streets or highways (30.8%), and high-energy falls and non-fall other injuries had relatively larger shares in recreation or sports settings (19.6% and 17.4%, respectively) (Figure 4A). Although most cases in every group involved neither vertebral nor hip co-incident fracture, vertebral fractures were proportionally most common in high-energy falls (28.9%), while hip fractures were most common in motor vehicle or transport injuries (12.7%) (Figure 4B). Emergency department disposition patterns also varied by mechanism. High-energy falls were most often treated and released (62.0%); motor vehicle or transport injuries had the highest proportion of admission or hospitalization (54.6%); low-energy falls were nearly evenly divided between treated and released (46.8%) and admitted or hospitalized (46.0%), and non-fall other injuries were more often treated and released (52.5%) than admitted or hospitalized (39.8%) (Figure 4C).

In a survey-aware multinomial logistic regression model of sacral fracture etiology, low-energy falls were used as the reference category while accounting for NEISS weights, strata, and PSU. Increasing age was associated with lower relative odds of all alternative etiologies, including high-energy falls (OR 0.697 per 10-year increase, 95% CI 0.630 to 0.771, *p* < 0.001), motor vehicle or transport injuries (OR 0.775, 95% CI 0.723 to 0.832, *p* < 0.001), and non-fall other injuries (OR 0.821, 95% CI 0.730 to 0.923, *p* = 0.001), each relative to low-energy falls.

Male sex was associated with higher relative odds of motor vehicle or transport injury compared with low-energy falls (OR 1.456, 95% CI 1.121 to 1.892, *p* = 0.005), but was not significantly associated with high-energy falls (OR 1.415, 95% CI 0.895 to 2.237, *p* = 0.138) or non-fall other injuries (OR 0.818, 95% CI 0.513 to 1.302, *p* = 0.397). Calendar year was not significantly associated with high-energy falls (OR 0.991, 95% CI 0.942 to 1.043, *p* = 0.734), motor vehicle or transport injuries (OR 1.046, 95% CI 0.992 to 1.103, *p* = 0.099), or non-fall other injuries (OR 0.979, 95% CI 0.922 to 1.039, *p* = 0.480), each relative to low-energy falls.

## 4. Discussion

### 4.1. Principal Findings

NEISS identified a rising national ED burden of SFx from 2003 to 2022. Visits were predominantly among older female patients after low-energy falls, with injuries most often occurring at home. Males were more represented in high-energy falls and motor vehicle/transport injuries, and motor vehicle/transport injuries had the highest admission or hospitalization proportion. Concurrent fracture patterns did not differ significantly by mechanism, although vertebral fractures were proportionally most common in high-energy falls and hip fractures in motor vehicle/transport injuries.

### 4.2. Rising Emergency Department Burden and Distinct Mechanistic Patterns in Sacral Fracture Presentations

The rise in SFx-related ED visits indicated a growing acute-care burden, although NEISS could not distinguish true changes in fracture occurrence from changing care-seeking or diagnostic practices. Demographic aging likely contributed, given the increasing concentration of pelvic fragility fractures in older adults as osteoporosis and fall risk accumulated [9]. Sacral injuries were often occult on radiographs and could present with nonspecific low back, buttock, or pelvic pain, which may have influenced recognition over time [10]. The steeper increase among women was consistent with sex differences in osteoporosis and fragility fracture risk and with frameworks positioning sacral insufficiency injury within the pelvic fragility spectrum [9]. Rather than establishing the cause of the temporal increase, these findings demonstrate that SFx account for a progressively larger share of ED presentations over time, particularly among older women, underscoring the growing relevance of fall prevention, bone health assessment, and ED disposition planning.

SFx presentations showed two descriptive patterns rather than discrete biologic categories: low-energy falls among older women, consistent with pelvic fragility literature [9], and higher-energy or transport-related injuries among younger patients and males, consistent with sex differences in transport injury epidemiology [11]. Because NEISS lacked fracture morphology, osteoporosis status, and injury severity measures, these patterns should be interpreted as ED presentation phenotypes rather than causal fracture subtypes.

### 4.3. Fall Prevention, Opportunistic Imaging, and Bone Health

Low-energy, home-based falls in older adults represented the dominant pattern underlying national SFx ED volume, suggesting that many of these presentations occur within a broader fragility context rather than as isolated injuries. Prior work has described pelvic fragility and sacral insufficiency injuries as part of a geriatric syndrome associated with impaired mobility, fall predisposition, and osteoporosis risk [9]. The age and sex distribution observed in our cohort is also consistent with the higher burden of osteoporosis and fragility fracture risk among older women [12]. Although NEISS does not provide bone density, osteoporosis status, or medication data, these findings support considering SFx in older adults as a potential marker of broader fall and bone health vulnerability.

Many sacral fractures are identified on CT or MRI obtained during evaluation of falls or persistent pelvic pain, which creates a potential opportunity to use already obtained imaging to support bone health assessment. Opportunistic CT approaches estimate bone density by measuring trabecular attenuation in a lumbar vertebral body, commonly L1, on routine CT, and these measures correlate with DXA-derived bone mineral density and fracture risk [13,14,15]. Prior work suggests that very low L1 trabecular attenuation, approximately ≤110 Hounsfield units (HU), is strongly suggestive of osteoporosis, whereas higher values, approximately ≥160 HU, make osteoporosis less likely [16,17]. Simplified thresholds were also proposed, with >150 HU generally reassuring and <120 HU, especially <100 HU, raising concern for low bone density [18]. Although such approaches are not directly evaluated in NEISS, they illustrate one possible pathway for secondary prevention after an ED sacral fracture presentation.

ED-based follow-through after discharge or admission could include fall-risk assessment, mobility planning, and osteoporosis evaluation or referral, especially because many SFx patients were discharged. Fracture liaison services and Own the Bone^®^ offered systems-based models for post-fracture bone health evaluation and treatment [19,20]. Targeted prevention was further supported by evidence that orthostatic hypotension, medication burden, impaired balance, and low exercise exposure were modifiable fall contributors [21,22,23].

### 4.4. Mechanism-Guided ED Triage and Resource Planning

Mechanism-specific differences in concurrent fracture patterns and ED disposition suggest that ED triage, imaging strategies, and downstream care pathways should be tailored by subgroup. Sacral injuries can be occult on plain radiography, and delayed recognition may prolong pain, immobility, and repeated healthcare utilization; accordingly, symptom- and mechanism-informed escalation to cross-sectional imaging may be particularly important in older fall patients with persistent posterior pelvic pain or inability to mobilize [24]. Conversely, higher-energy falls and transport injuries align with higher admission or transfer rates and may justify earlier multidisciplinary trauma systems and broader co-injury screening, particularly in younger patients and males. From a public health perspective, the rising proportion of higher-energy mechanisms over time underscores the need for injury-prevention strategies beyond geriatric falls, including transport-related and environmental exposure prevention, where sex differences in exposure patterns and crash biomechanics may contribute to differing injury distributions [11]. Future studies linking ED presentations to longitudinal clinical data could clarify mechanism-specific outcomes, refine risk stratification, and characterize downstream consequences of the rising SFx ED burden across mechanism groups.

### 4.5. Downstream Care Implications

Although NEISS does not provide fracture morphology, instability patterns, neurologic findings, or treatment details, the observed mechanism-specific differences still have downstream clinical relevance. Prior fragility fracture frameworks suggest that sacral injuries in older adults can contribute to pain, immobility, and loss of independence, while higher-energy sacral injuries may occur in the setting of more complex posterior pelvic ring trauma and broader co-injury burden [25,26]. When procedural management is required, contemporary care increasingly includes less invasive posterior stabilization strategies across both fragility-related [27] and higher-energy injury settings [28,29]. However, the present dataset cannot determine operative indications, technique selection, or comparative treatment outcomes. Accordingly, the main implication of our findings is not to define operative management, but rather to support timely recognition, mechanism-informed triage, and appropriate referral pathways across ED, trauma, and spine care systems.

### 4.6. Study Strengths, Limitations, and Future Directions

NEISS provided a nationally representative sample of U.S. ED visits over 20 years, captured patients treated and released rather than only hospitalized cohorts, and supported national estimates using complex survey weights [30,31]. Narrative confirmation improved diagnostic specificity for sacral involvement, which was important because sacral injuries may be overlooked without targeted evaluation or advanced imaging [24,25,26,27,28,29,30,31,32]. Prespecified abstraction rules and independent dual review strengthened reproducibility, although mechanism assignment remained limited by available coded fields and narratives [30,31]. Several limitations should be considered. NEISS captures U.S. emergency department encounters rather than unique incident fractures. Therefore, the population-based rates reported in this study represent sacral fracture ED visits rather than true fracture incidence. Repeat ED encounters for the same injury may be represented more than once, while fractures managed outside the ED or not recognized during the indexed encounter are not captured [30]. Although narrative review improved ascertainment, documentation and coding limitations could have caused misclassification of mechanism, location, and concurrent injuries, particularly because SFx could be occult on radiographs and under-detected without cross-sectional imaging [24,32]. Because candidate cases were identified using prespecified fracture and body-part codes, sacral fractures assigned to body-part categories outside the initial search may not have been captured, potentially resulting in under-ascertainment. NEISS also lacked fracture morphology, osteoporosis status, comorbidities, treatment details, neurologic findings, and longitudinal outcomes, limiting assessment of injury severity, management, and recovery. In addition, race data were missing in 17.8% of visits, which limited assessment of racial differences in presentation, mechanism, and disposition and reduced our ability to evaluate disparities using NEISS alone. Future work should link ED presentations to longitudinal data sources to quantify 30-day and 1-year outcomes, repeat ED use, subsequent fragility fractures, mortality, and care transitions after discharge versus admission. Imaging-confirmed validation studies could refine NEISS case capture, mechanism classification, and co-injury abstraction. Pragmatic studies should test ED-facing fragility pathways triggered by SFx while preserving rapid co-injury screening for higher-energy presentations.

## 5. Conclusions

In this national analysis of NEISS emergency department encounters from 2003 to 2022, sacral fracture (SFx) ED visits increased sharply over time, with the rise more pronounced among women and accompanied by an increase in mean age at presentation. Mechanism patterns showed two broad descriptive presentations: low-energy, home-based falls in older adults comprised the dominant pathway underlying national ED burden, while higher-energy falls and motor vehicle or transport-related injuries were more common in younger patients and males and were associated with higher rates of admission or transfer. Concurrent fractures, particularly vertebral and hip fractures, were common and varied by mechanism, underscoring that SFx often occur within a broader injury or fragility context rather than as isolated events. Collectively, these findings position SFx as an increasingly important ED-facing condition with prevention and systems-planning implications, including stronger integration of fall-risk reduction and bone-health evaluation pathways for older adults, alongside mechanism-informed triage and referral for higher-energy presentations.

## Figures and Tables

**Table 1:** Demographics and injury etiology across all patients.

Variable	Overall
Unweighted Count	1747 (100.0%)
Weighted Count	72,215 (100.0%)
Age Mean (SD)	61.1 (23.7)
Sex (%)	
Male	21,695 (30.0%)
Female	50,520 (70.0%)
Race (%)	
White	52,776 (73.1%)
Black/African American	3012 (4.2%)
Asian	963 (1.3%)
American Indian/Alaska Native	2247 (3.1%)
Native Hawaiian/Pacific Islander	264 (0.4%)
Other	89 (0.1%)
Not Specified	12,864 (17.8%)
Fall: high-energy	4653 (6.4%)
Fall: low-energy	49,011 (67.9%)
Non-fall: motor vehicle/transport	12,446 (17.2%)
Non-fall: other	6106 (8.5%)
Unweighted Count	1747 (100.0%)

**Table 2:** Comparison of demographics across injury etiology.

Variable	Fall: High-Energy	Fall: Low-Energy	Non-Fall: Motor Vehicle/Transport	Non-Fall: Other	*p*-Value
Unweighted Count	106 (100.0%)	1210 (100.0%)	291 (100.0%)	146 (100.0%)	
Weighted Count	4653 (100.0%)	49,011 (100.0%)	12,446 (100.0%)	6106 (100.0%)	
Age Mean (SD)	44.9 (20.7)	65.7 (22.2)	52.4 (24.0)	54.7 (25.0)	<0.001
Sex (%)					<0.001
Male	1914 (41.1%)	13,150 (26.8%)	5021 (40.3%)	1610 (26.4%)	
Female	2738 (58.9%)	35,861 (73.2%)	7425 (59.7%)	4496 (73.6%)	
Race (%)					0.126
White	3473 (74.6%)	35,863 (73.2%)	8854 (71.1%)	4586 (75.1%)	
Black/African American	240 (5.2%)	1960 (4.0%)	675 (5.4%)	136 (2.2%)	
Asian	33 (0.7%)	807 (1.6%)	104 (0.8%)	18 (0.3%)	
American Indian/Alaska Native	64 (1.4%)	1320 (2.7%)	675 (5.4%)	189 (3.1%)	
Native Hawaiian/Pacific Islander	0 (0.0%)	158 (0.3%)	16 (0.1%)	90 (1.5%)	
Other	0 (0.0%)	24 (0.0%)	65 (0.5%)	0 (0.0%)	
Not Specified	843 (18.1%)	8878 (18.1%)	2056 (16.5%)	1088 (17.8%)	

**Table 3:** Comparison of setting, co-incident fractures, and disposition across all patients.

Variable	Overall
Unweighted Count	1747 (100.0%)
Weighted Count	72,215 (100.0%)
Setting	
Home	36,467 (50.5%)
Farm/Ranch	225 (0.3%)
Street/Highway	4093 (5.7%)
Other Public Property	7867 (10.9%)
Mobile/Manufactured Home	98 (0.1%)
School/Daycare	349 (0.5%)
Place of Recreation/Sports	5551 (7.7%)
Not Specified	17,565 (24.3%)
Concurrent Fractures	
Vertebral Fracture	15,814 (21.9%)
Hip Fracture	7682 (10.6%)
Both Vertebral and Hip Fractures	1493 (2.1%)
Neither	50,212 (69.5%)
Disposition	
Treated/Examined and Released	33,746 (46.7%)
Treated and Transferred	3510 (4.9%)
Treated and Admitted/Hospitalized	33,019 (45.7%)
Held for Observation	1802 (2.5%)
Left Without Being Seen	84 (0.1%)

**Table 4:** Comparison of setting, co-incident fractures, and disposition across etiology.

Variable	Fall: High-Energy	Fall: Low-Energy	Non-Fall: Motor Vehicle/Transport	Non-Fall: Other	*p*-Value
Unweighted Count	106 (100.0%)	1210 (100.0%)	291 (100.0%)	146 (100.0%)	
Weighted Count	4653 (100.0%)	49,011 (100.0%)	12,446 (100.0%)	6106 (100.0%)	
Setting					<0.001
Home	2167 (46.6%)	29,445 (60.1%)	2652 (21.3%)	2203 (36.1%)	
Farm/Ranch	137 (2.9%)	73 (0.1%)	0 (0.0%)	15 (0.3%)	
Street/Highway	0 (0.0%)	219 (0.4%)	3839 (30.8%)	34 (0.6%)	
Other Public Property	73 (1.6%)	6312 (12.9%)	820 (6.6%)	663 (10.9%)	
Mobile/Manufactured Home	0 (0.0%)	98 (0.2%)	0 (0.0%)	0 (0.0%)	
School/Daycare	0 (0.0%)	327 (0.7%)	22 (0.2%)	0 (0.0%)	
Place of Recreation/Sports	911 (19.6%)	2225 (4.5%)	1355 (10.9%)	1060 (17.4%)	
Not Specified	1365 (29.3%)	10,312 (21.0%)	3758 (30.2%)	2130 (34.9%)	
Concurrent Fractures					0.167
Vertebral Fracture	1345 (28.9%)	9092 (18.6%)	2576 (20.7%)	1308 (21.4%)	
Hip Fracture	119 (2.6%)	4110 (8.4%)	1586 (12.7%)	375 (6.1%)	
Both Vertebral and Hip Fractures	0 (0.0%)	1094 (2.2%)	261 (2.1%)	138 (2.3%)	
Neither	3188 (68.5%)	34,715 (70.8%)	8023 (64.5%)	4285 (70.2%)	
Disposition					0.01
Treated/Examined and Released	2887 (62.0%)	22,951 (46.8%)	4701 (37.8%)	3207 (52.5%)	
Treated and Transferred	356 (7.6%)	2111 (4.3%)	715 (5.7%)	328 (5.4%)	
Treated and Admitted/Hospitalized	1278 (27.5%)	22,521 (46.0%)	6790 (54.6%)	2430 (39.8%)	
Held for Observation	77 (1.7%)	1412 (2.9%)	239 (1.9%)	73 (1.2%)	
Left Without Being Seen	0 (0.0%)	16 (0.0%)	0 (0.0%)	67 (1.1%)	

## Data Availability

Data is publicly available at https://www.cpsc.gov/Research--Statistics/NEISS-Injury-Data. (accessed on 14 November 2025).

## Abbreviations

The following abbreviations are used in this manuscript:

SFx	Sacral Fracture
NEISS	National Electronic Injury Surveillance System
